# Delta Opioid Receptors and Enkephalinergic Signaling within Locus Coeruleus Promote Stress Resilience

**DOI:** 10.3390/brainsci12070860

**Published:** 2022-06-29

**Authors:** John A. Tkaczynski, Olga Borodovitsyna, Daniel J. Chandler

**Affiliations:** Department of Cell Biology and Neuroscience, Rowan University School of Osteopathic Medicine, 42 East Laurel Road, Stratford, NJ 08084, USA; tkaczy28@rowan.edu (J.A.T.); borodovio3@rowan.edu (O.B.)

**Keywords:** locus coeruleus, stress, enkephalin, delta opioid receptors, anxiety-like behavior

## Abstract

The noradrenergic nucleus locus coeruleus is a key component of the stress circuitry of the brain. During stress, the neuropeptide corticotropin-releasing factor (CRF) is secreted onto LC, increasing LC output and norepinephrine concentration in the brain, which is thought to promote anxiety-like behavior. LC is also innervated by several structures that synthesize and release the endogenous opioid peptide enkephalin onto LC upon stressor termination. While the role of CRF neurotransmission within LC in mediating anxiety-like behavior and the behavioral response to stress has been well characterized, the role of enkephalinergic signaling at LC-expressed δ-opioid receptors has been comparatively understudied. We have previously shown that acute stressor exposure increases LC activity and anxiety-like behavior for at least one week. Here, we extend these findings by showing that these effects may be mediated at least in part through stress-induced downregulation of DORs within LC. Furthermore, overexpression of DORs in LC blocks the effects of stress on both LC firing properties and anxiety-like behavior. In addition, intra-LC infusions of enkephalin blocked stress-induced freezing behavior and promoted conditioned place preference. These findings indicate that enkephalinergic neurotransmission at DORs within LC is an important component of the behavioral response to stress and may drive reward-related behavior as well.

## 1. Introduction

The locus coeruleus (LC) is a nucleus in the brainstem that serves as the primary site of norepinephrine (NE) synthesis in the central nervous system and has classically been considered to be a relatively homogeneous structure comprising neurons that project broadly throughout the neuraxis through a network of highly divergent axons [1,2]. Consistent with these anatomical reports, the LC has been implicated in a variety of functions, such as the regulation of wakefulness and sleep [3], memory consolidation [4], sensory signal processing [5], cognition [6,7], and the behavioral response to stressful stimuli [8,9]. The role of the LC in this context has been particularly well characterized. An extensive body of literature from the past 40 years has shown that the LC is densely innervated by a number of corticotropin-releasing factor (CRF)-containing structures in the brain, including the paraventricular nucleus of the hypothalamus (PVN), bed nucleus of stria terminals (BNST), central nucleus of the amygdala (CeA), and Barrington’s nucleus [10,11,12,13]. During periods of stress, CRF is released onto the LC, where in the short term it exerts a stimulatory effect via CRFR1, a Gs-coupled receptor that, through the actions of protein kinase A PKA, reduces potassium conductance, leading to cellular depolarization and increased tonic firing [14], thereby promoting release of NE into the forebrain [15]. Behaviorally, it has been shown that intra-LC release of CRF increases LC output and is causally responsible for the generation of anxiety-like behavior, both learned and in real time [13]. However, evidence suggests that CRF also promotes long-term cellular adaptations through regulation of transcription [16,17]. In line with these reports, previous observations from our laboratory have shown that an acute stressor increases LC tonic discharge and excitability, effects which persist for at least one week [18], indicating that stressor exposure precipitated long-lasting cellular adaptations [9]. Importantly, these persistent physiological changes were also accompanied by increased anxiety-like behavior which also endured for at least one week [18]. These findings are consistent with prior reports of persistent effects of acute traumatic-like stress on hyperarousal in both mice and rats [19,20].

These findings show that LC is an important anatomical substrate that can be targeted for the modulation of anxiety-like behavior pre-clinically, and potentially for the treatment of anxiety disorders within clinical patient populations. Despite this, CRF receptor antagonists have generally not proven effective as therapeutics for the treatment of anxiety disorders in clinical trials [21]. However, in addition to the CRF-positive input from several stress-related structures, it is also known that LC neurons express inhibitory µ-opioid receptors (MORs) and δ-opioid receptors (DORs) [22,23,24,25,26,27], which are activated by the endogenous opioid enkephalin released from several afferent nuclei such as the nucleus prepositus hypoglossi and the nucleus paragigantocellularis [12,28,29,30]. Enkephalins are a class of peptides of 5–8 amino acids in length derived from the proenkephalin gene and prodynorphin gene; they are named after the terminal amino acid (Met-enkephalin and Leu-enkephalin) and have a high affinity for MOR, but even higher for DORs [31]. Importantly, these afferents are engaged during stressor termination, activating Gi-coupled endogenous opioid receptors in LC, hyperpolarizing its neurons to counteract the pro-stress effects of CRF signaling [32,33], and presumably facilitating a return to a non-anxiety-like behavioral phenotype [25]. Interestingly, the CRF-positive CeA projection to LC and the enkephalin-positive PGi projection to LC seem to become engaged under different behavioral circumstances. Specifically, it has been shown that in the resident intruder model of psychosocial stress, rats that have a longer latency to defeat and are therefore more stress resilient show increased c-Fos expression in the enkephalin-positive PGi neurons that project to LC. Conversely, in animals with a short latency to defeat and are therefore more stress susceptible, there is increased expression of cFos in CRF-positive CeA neurons that project to LC [30]. These observations, in conjunction with reports that optogenetic release of CRF from CeA terminals in LC drives anxiety-like behavior [13], indicate that these distinct pathways promote specific behavioral phenotypes in response to stress. This is also indirectly corroborated by studies that show that genetic and pharmacological manipulations of both enkephalin and DORs not specific to LC also affect anxiety-like behaviors [34,35,36,37]. However, the role of enkephalinergic neurotransmission at DORs expressed by LC specifically within the context of stress and behavior has not been well characterized. Therefore, we sought to determine how stress and DOR expression in the LC interact to modulate anxiety-like behavior. Additionally, because LC hyperactivity is aversive, we also aimed to show that enkephalinergic signaling in LC not only reduces stress-induced anxiety-like behavior but also promotes reward-related behavior by suppressing LC. Here, we show that stress decreases DOR expression in LC, and that viral-genetic LC-specific DOR overexpression prevents stress-induced changes in LC physiological properties and anxiety-like behaviors in the elevated plus maze (EPM) and open field test (OFT). Furthermore, enkephalin infusions in the LC also led to decreased anxiety-like behavior in the OFT and promoted conditioned place preference, indicating a novel role for this neuropeptide in anxiety-like and motivated behavior.

## 2. Materials and Methods

### 2.1. Subjects

Male Sprague Dawley rats (Taconic Farms) were housed 2–3 per cage on a 12 h reverse light–dark cycle (lights on at 9:00 p.m.) with access to standard rat chow and water ad libitum. Animal protocols were approved by the Rowan University Institutional Animal Care and Use Committee and were conducted in accordance with National Institutes of Health Guide for the Care and Use of Laboratory Animals.

### 2.2. Stressor Exposure

Stressor exposure was performed as previously described at 6–7 weeks of age [18]. Rats were habituated to the experimenter by gentle handling for 10–15 min per day for three days before behavioral testing began. Subjects were also habituated to a plastic enclosure in which stress or control conditions took place. Stress and control conditions, as well as behavioral testing, took place in a dimly lit room. Acute stress was induced by placing rats in a rodent restrainer (Harvard Apparatus, Holliston, MA, USA) for 15 min which was placed inside of a sealed anesthesia induction chamber connected by silicone tubing to an aquarium pump. A small plastic cylinder was placed in-line with the tubing. A 2.5 cm × 2.5 cm piece of filter paper was placed inside of the tube and saturated with 100 μL predator odor (2,4,5-trimethylthiazole, TMT, Sigma-Aldrich, St. Louis, MO, USA). Odor delivery was achieved by turning on the aquarium pump so that the air forced through the tubing carried the odor into the airtight odor exposure chamber. Control animals were placed in an identical bell chamber for 15 min, but they were not restrained and no odor was delivered.

### 2.3. Elevated plus Maze

The elevated plus maze (EPM) consisted of a plus-shaped black plexiglass apparatus elevated 76 cm off the ground with two sets of opposing arms (each arm = 40 cm in length) meeting in a central 10 cm × 10 cm area. Two opposing arms have vertical walls extending 30 cm from the floor of the maze, while the other two arms do not have walls. Rats were allowed to explore the maze for 10 min. Their activity was filmed with an infrared camera situated above the maze connected to a Lenovo ThinkCentre M700 PC. At the conclusion of each test, rats were either returned to their home cage for a week, or sacrificed for electrophysiological recordings. The maze was cleaned with 10% bleach between each test. Open arm time, time freezing, time mobile, and average speed were scored using AnyMaze Version 7.14 behavioral tracking software (Stoelting, Wood Dale, IL, USA). The onset of freezing episodes was defined by a period of 1 s without motion, and were terminated when motion was again detected.

### 2.4. Open Field Test

The open field test (OFT) consisted of a 90 cm × 90 cm × 30 cm black plexiglass box. Rats were allowed to explore the apparatus for 10 min, during which their activity was filmed with an infrared camera situated above the maze connected to a Lenovo ThinkCentre M700 PC. At the conclusion of the test, rats were sacrificed for either electrophysiological recordings or histology. The apparatus was cleaned with 10% bleach between each test. Center time and time freezing were scored using AnyMaze behavioral tracking software (Stoelting, RRID SCR_014289). The onset of freezing episodes was defined by a period of 1 s without motion, and were terminated when motion was again detected.

### 2.5. Viral Injections

Surgical procedures were performed according to a standard protocol as we have described previously [38]. Briefly, rats were deeply anesthetized through isoflurane inhalation (4% induction, 1–2% maintenance) and placed in a stereotaxic frame. Rats for overexpression studies were approximately 4 weeks of age at the time of surgery. This allowed additional survival time for virally transduced rats to permit transgene expression. Rats received a single injection 0.3 µL of DOR overexpression vector (AAV-PRSx8-oprd1, Applied Biological Materials) or control vector (AAV-PRSx8-mCherry, kindly provided by Dr. Elena Vazey) on each side of the brain using the following coordinates from lambda to target LC: AP = −1.2 mm; ML = ±1.4 mm; DV = −6.6 mm. All injections were performed using a 1.0 µL Hamilton Neuros syringe mounted in a World Precision Instruments stereotax-mounted injection pump at a flow rate of 50 nL/min. Syringes remained in place for 10 min before removal. Craniotomies were filled with sterile bone wax, and the incision was closed with wound clips. Following surgery, rats underwent a three week recovery to permit transgene expression. Injection sites were confirmed histologically after the completion of all experiments.

### 2.6. RT-PCR

All tools, materials, and instruments were autoclaved and treated with RNAse Zap (Invitrogen, Waltham, MA, USA) prior to use to prevent degradation of RNA. Rats were deeply anesthetized with 4% isoflurane and rapidly decapitated. Brains were extracted and blocked coronally to a piece of tissue containing cerebellum and pons < 0.5 mm in length. This piece of tissue was placed in 1.8 mL RNALater (Invitrogen) at 4 °C for 24 h, then placed in a new dry vial followed by long-term storage at −20 °C. Brain blocks containing LC were attached to a cryostat mounting block with tissue freezing medium (Triangle Biomedical Sciences; Durham, NC, USA) at −30 °C and placed in a cryostat. The brain was trimmed to approximately 1.5 mm in length to contain the full rostrocaudal extent of LC. A 1 mm trephine was then used to collect bilateral punches of the area directly lateral to the fourth ventricle to collect LC. LC punches were collected in 350 µL RLT lysis buffer (Qiagen, Hilden, Germany) and homogenized using a glass Dounce homogenizer.

Total RNA was extracted from each LC tissue punch using a Qiagen RNeasy Micro Kit according to manufacturer instructions to produce 14 μL samples (*n* = 5/group). RNA concentration and purity within each sample was assessed by using 1 μL from each sample in a NanoDrop spectrophotometer (Thermo Scientific, Waltham, MA, USA). A TaqMan Reverse Transcription Reagent kit (Invitrogen) was used according to manufacturer instructions to produce and amplify ten 50 μL samples of cDNA for each animal in a BioRad DNA Engine. Samples were stored at −20 °C until further use in RT-PCR experiments. GAPDH was used as a housekeeping gene. In each experiment, individual wells contained 10 μL reaction mixture (consisting of 10 μL TaqMan 2X Master Mix, 4 μL DEPC water, 1 μL 20x primers, plus 5 μL cDNA per sample). Individual ΔΔCt experiments were carried out using software in conjunction with RT-PCR at Mastercycler EP Realplex (Eppendorf, Hamburg, Germany) to quantify relative expression of oprd1 mRNA. Fluorescence baselines and thresholds were manually set for each experiment. Threshold cycle (Ct) values were measured from each sample, and a mean Ct value was calculated from all samples. This same mean value was then subtracted from each individual ΔCt value obtained from each sample to produce an individual ΔΔCt value for each sample. These values were then used to generate relative quantity = 2^−ΔΔCt^ for each sample. This relative quantity for each sample was then divided by the mean control population relative quantity such that the mean of the control group was equal to 1. Therefore, each relative quantity for each sample represents a fold-change from the mean control population.

### 2.7. Electrophysiological Recordings

Rats were deeply anesthetized with an intraperitoneal injection of Euthasol (100 mg/kg, Virbac) and transcardially perfused with 60 mL ice-cold oxygenated artificial cerebrospinal fluid (aCSF) of the following composition (in mM): NaCl, 126; KCl, 2.5; CaCl_2_, 2.4; NaH_2_PO_4_, 1.2; MgCl_2_, 1.3; NaHCO_3_, 25; D-glucose, 11. Rats were then rapidly decapitated and the skull was removed so that gross coronal cuts could be made at the level of the medulla and the pineal gland; the resulting block of brain tissue was then extracted from the skull and transferred to 30 mL of ice-cold oxygenated sucrose-aCSF of the following composition (in mM): sucrose, 58.4; NaCl, 85; KCl, 2.5; CaCl_2_, 2.4; NaH_2_PO_4_, 1.2; MgCl_2_, 1.3; NaHCO_3_, 25. The brain remained in the sucrose-aCSF for 1–2 min, after which it was transferred to a piece of filter paper saturated with ice-cold oxygenated sucrose aCSF, and the lateral edges of the brain were trimmed off. The dorsal aspect of the brain was then glued to the stage of a Compresstome VF-300-0Z tissue slicer, embedded in agarose, and submerged in ice-cold oxygenated sucrose aCSF; then, 200 μM thick horizontal sections were cut at a speed of 0.1 mm/s with an amplitude of 1.0 mm. Sections containing LC (typically 3–4 per animal) were transferred to a holding incubator containing ~300 mL aCSF continuously bubbled with 95% O_2_/5% CO_2_ maintained at 35.5 °C and supported by nylon mesh for 1 h. After 1 h, the holding incubator was maintained at room temperature.

Brain slices were individually transferred to a recording chamber which was continuously superfused at 1.5–2 mL/min with oxygenated aCSF maintained at 37 °C by a Warner Instrument Corporation in-line heater (model 60-01013). LC was visualized as a semi-translucent crescent-shaped region located lateral to the fourth ventricle at 5× magnification using an Olympus BX51WI fixed-stage upright microscope with differential interference contrast and an infrared filter. Individual LC neurons were visualized with a 40× immersion lens and QImaging Rolera Bolt camera connected to a Lenovo ThinkCentre M700 desktop computer using QCapture Pro software. Neurons were approached with patch electrodes (resistance = 5–10 MΩ) controlled with Sutter MPC-200 manipulators. Electrodes were filled with intracellular solution of the following composition (in mM): KCl, 20; K-gluconate, 120; MgCl_2_, 2; EGTA, 0.2; HEPES, 10; Na_2_ATP, 2. After a GΩ seal was established between the pipette and neuronal membrane, the membrane was ruptured, and neurons were allowed to equilibrate for 2–3 min prior to data acquisition. Whole-cell recordings were made with a MultiClamp 700B amplifier, Digidata 1550B digitizer equipped with two HumSilencer channels, and ClampEx 10.6 software. Electrophysiological data were analyzed using a two-factor design (treatment: control vs. stress, virus: AAV-PRSx-oprd1 vs. AAV-PRSx8-mCherry). To assess membrane properties in current clamp mode, spontaneous activity was recorded for 60 s without any input and the average firing rate was calculated. Electrophysiological data were analyzed with Molecular Devices ClampFit 10.6 software.

### 2.8. Bilateral Cannulae Implantation

Rats were deeply anesthetized through isoflurane inhalation (4% induction, 1–2% maintenance) and placed in a stereotaxic frame. Rats were approximately 5–6 weeks of age at the time of surgery to allow a week of post-operative recovery before stress or control conditions took place. Rats were implanted with bilateral microinfusion cannulae (Plastics One) in LC using the following coordinates from lambda to target LC: AP = −1.2 mm, ML = ±1.4 mm, DV = −6.6 mm. Several #0–80 screws (0.25” length) were also set in the skull, and GC FujiCEM2 dental cement was used to secure implants. Internal dummy cannulae were then inserted into the implanted microinfusion guide cannulae and were covered by a dust cap. Rats were then singly housed and returned to their home cages for a one-week recovery to permit transgene expression. Injection sites were confirmed histologically after the completion of all experiments. Infusions of saline (1 µL per side) or Leu-enkephalin (10 pg dissolved in 1 µL saline per side) were performed while awake using a kD Scientific double-barrel syringe pump fitted with two 5 µL Hamilton 7000-series syringes that connected to the infusion cannulae with PE100 tubing. Infusions occurred at a flow rate of 0.5 µL/min. In experiments used to determine the effects of intra-LC enkephalin on open field behavior, infusions took place immediately after stress or control conditions and immediately before being placed in the OFT apparatus. Implant sites were confirmed histologically after the completion of all experiments.

### 2.9. Conditioned Place Preference

The CPP apparatus consisted of two 16” × 16” × 16” acrylic boxes joined by a 4” × 16” × 16” compartment. One of the larger compartments had black vertical stripes on light gray walls, and the other had large black polka dots on light gray walls. Rats were habituated to the experimenter by gentle handling for 10–15 min per day for three days before conditioning began. For the conditioning phase, each day for eight days, all rats received bilateral 1 µL infusions of saline before being placed in and confined to the chamber with black stripes (unconditioned side) for 30 min. Two hours later, rats received bilateral 1 µL infusions of either saline (*n* = 5) or saline containing 10 pg Leu-enkephalin (*n* = 6) before being placed in and confined to the chamber with the black polka dots (conditioned side). One day after the final day of conditioning at 10 weeks of age, rats were placed in the small neutral chamber and were allowed to freely explore the apparatus for 15 min. Tests were filmed and analyzed with AnyMaze software. Preference for the conditioned chamber was calculated as the difference between the amount of time spent in the conditioned side and the amount of time spent in the unconditioned side.

### 2.10. Experimental Design and Statistical Analysis

Statistical analyses were performed with GraphPad Prism version 8.4.1 (San Diego, CA, USA). All data sets were tested for normality and homogeneity of variance. Those that satisfied both of these requirements underwent parametric testing as indicated in the text, while those that did not were subject to nonparametric statistical testing as described in the results section. Data are presented as mean ± SEM.

## 3. Results

### 3.1. Stressor Exposure Increases Anxiety-Like Behavior and Decreases LC DOR Expression

**Figure 1:**
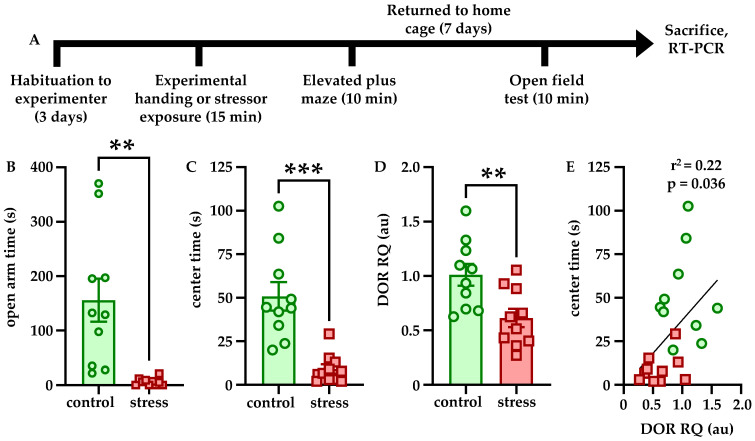
**Stress increases anxiety-like behavior and decreases LC DOR expression.** The experimental timeline is shown in (**A**). Rats were habituated to handling by the experimenter for 3 days but were naïve to the behavioral apparatus when testing began. Fifteen minutes of combined restraint and predator odor exposure increased anxiety-like behavior in the EPM immediately (**B**) and in the OFT one week later (**C**). Stressor exposure was also associated with decreased DOR expression (**D**) which significantly correlated with center time in the OFT (**E**). ** *p* < 0.01; *** *p* < 0.001.

Effects of stress on anxiety-like behavior and DOR expression were determined according to the experimental timeline shown in Figure 1A. Immediately after stressor exposure or control conditions, anxiety-like behavior was tested in the EPM. Stressor exposure significantly decreased open arm time relative to control (t = 3.776, df = 18, *p* = 0.0014, Figure 1B). One week later, anxiety-like behavior was tested again in the OFT. Stressor exposure significantly decreased center time relative to controls (t = 4.815, df = 18, *p* = 0.0001, Figure 1C). Freezing behavior was not affected by stressor exposure at either time point (data not shown). After the final behavioral test, rats were sacrificed for RT-PCR quantification of DOR expression in LC. Stressor exposure was associated with a significant downregulation of DOR mRNA relative to controls (t = 3.045, df = 18, *p* = 0.0070, Figure 1D). Anxiety-like behavior in the OFT was significantly correlated with DOR expression at the time of sacrifice (r = 0.4710, r^2^ = 0.2219, *p* = 0.0361).

### 3.2. LC-Specific DOR Overexpression Blocks the Effects of Stress

**Figure 2:**
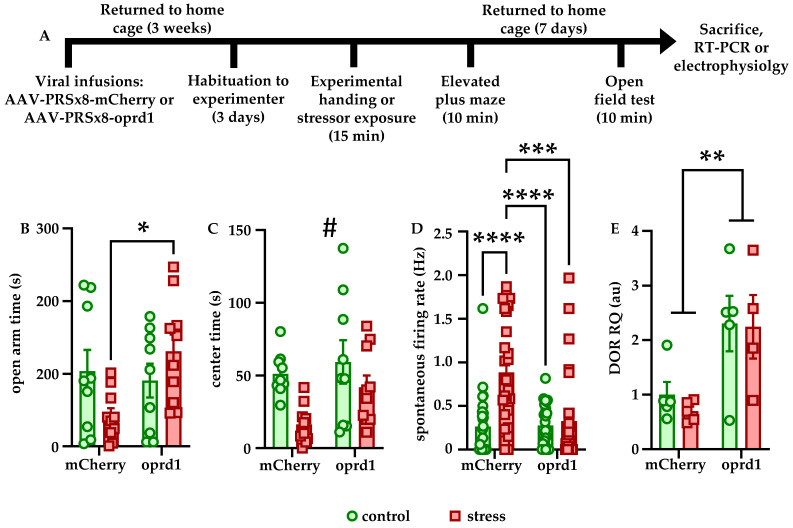
**LC-specific DOR overexpression blocks the effects of stress on anxiety-like behavior and LC firing.** The experimental timeline is shown in (**A**). Rats were habituated to handling by the experimenter for 3 days but were naïve to the behavioral apparatus when testing began. Stressed rats overexpressing DORs in LC spent significantly more time in the open arms of the EPM than stressed rats expressing mCherry in LC (**B**). One week after stressor exposure, stressed rats spent significantly less time in the center of the OFT, but this effect was more prominent in rats expressing mCherry than rats overexpressing DORs in LC (**C**). Within the mCherry group, stress significantly increased LC firing rate. In addition, LC neurons from stressed mCherry rats fired significantly faster than those from control or stressed DOR-overexpressing rats (**D**). DOR overexpression was validated using RT-PCR (**E**). * *p* < 0.05; ** *p* < 0.01; *** *p* < 0.001; **** *p* < 0.0001; # main effect of stress, *p* = 0.0055.

Because of the effects of stressor exposure on DOR expression and behavior, we sought to determine the effects of LC-specific DOR overexpression on these measures according to the experimental timeline in Figure 2A. Although the main effects of stress (F [1, 34] = 2.932, *p* = 0.0959) and expression (F [1, 34] = 0.2318, *p* = 0.6333) were not significant, there was a significant interaction between these two variables on open arm time in the EPM (F [1, 34] = 5.307, *p* = 0.0275). Bonferroni-corrected post hoc comparisons showed that stressed animals overexpressing DORs in LC spent significantly more time in the open arms than stressed animals expressing mCherry in LC (*p* = 0.0373), indicating a reduction in anxiety-like behavior (Figure 2B). One week later, anxiety-like behavior was again tested in the OFT. Although the main effects expression (F [1, 34] = 3.626, *p* = 0.0654) and the stress × expression interaction (F [1, 34] = 0.9781, *p* = 0.3297) were not significant, there was a significant main effect of stress on center time in the OFT (F [1, 34] = 8.775, *p* = 0.0055), indicating that stressed animals spent significantly less time in the center than control animals (Figure 2C). Freezing behavior was not affected by either stressor exposure or expression at either time point (data not shown).

Previous observations from our lab have shown that one week after stressor exposure, LC spontaneous firing rate also significantly increased relative to controls. To determine how DOR overexpression affected the physiological response to stress, animals were sacrificed immediately after the final behavioral test for LC whole-cell patch clamp recordings. The main effect of stress (F [1, 102] = 14.09, *p* = 0.0003), main effect of expression (F [1, 102] = 9.353, *p* = 0.0028), and the stress × interaction effect (F [1, 102] = 10.11, *p* = 0.002) were all highly significant. Bonferroni-corrected post hoc comparisons showed that within the mCherry expressing rats, stress significantly increased spontaneous firing rate (*p* < 0.0001). In addition, the firing rate of LC cells from stressed mCherry expressing rats was significantly higher than that of cells from both control (*p* < 0.0001) and stressed (*p* = 0.0002) DOR-overexpressing rats, indicating stress increases LC firing rate, but that DOR overexpression blocks this effect (Figure 2D). Finally, RT-PCR was used to validate that the AAV-PRSx8-oprd1 vector increased DOR mRNA expression in a small cohort of animals. Although the main effect of stress (F [1, 15] = 0.2219, *p* = 0.6444) and the stress × expression interaction effect (F [1, 15] = 0.1014, *p* = 0.7545) were not significant, there was a significant main effect of expression (F [1, 15] = 13.75, *p* = 0.0021) on DOR mRNA relative quantity (Figure 2E), indicating that the virus significantly increased DOR expression within LC.

### 3.3. Intra-LC Enkephalin Infusions Block Stress-Induced Freezing Behavior

**Figure 3:**
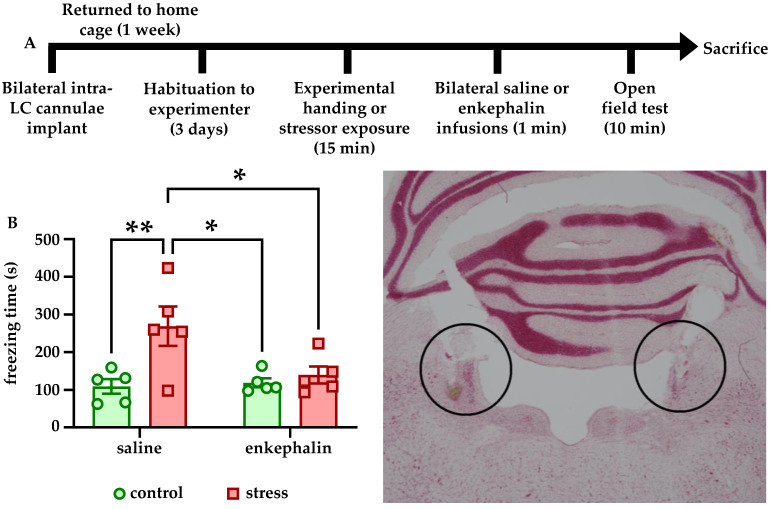
**Intra-LC infusions of enkephalin block stress-induced freezing.** The experimental timeline is shown in (**A**). Rats were habituated to handling by the experimenter for 3 days but were naïve to the behavioral apparatus when testing began. Rats that received saline infusions into LC froze in the OFT significantly more than control rats that received saline infusions, and more than control or stressed rats that received enkephalin infusions. Stressed rats that received enkephalin infusions did not freeze more than control rats that received enkephalin infusions (**B**). A representative photomicrograph showing bilateral cannulae placement within LC is shown in the bottom right. * *p* < 0.05; ** *p* < 0.01.

To determine how enkephalinergic signaling at LC-expressed opioid receptors affected the behavioral response to stress, animals were subject to the experimental timeline shown in Figure 3A. Immediately after stressor exposure or control conditions, rats received bilateral intra-LC infusions of 1 µL saline or saline containing 10 pg Leu-enkephalin, and were then placed immediately in the OFT. There were no effects of stress or enkephalin on center time in the OFT (data not shown). However, there was a significant main effect of stress (F [1, 16] = 8.639, *p* = 0.0096) and a significant stress × enkephalin interaction (F [1, 16] = 5.094, *p* = 0.0384) on freezing time. The main effect of enkephalin showed a trend towards, but did not reach, significance (F [1, 16] = 3.808, *p* = 0.0688). Bonferroni-corrected post hoc comparisons showed that stress significantly increased freezing in the saline-infused animals relative to control (*p* = 0.0099). In addition, stressed rats that were infused with saline froze significantly more than both stressed rats that were infused with enkephalin (*p* = 0.0401) and control rats that were infused with enkephalin (*p* = 0.0154, Figure 3B). These findings indicate that enkephalin infusions into LC blocked stress-induced freezing behavior. A representative image of the brain showing cannulae placement in LC is shown in Figure 3C.

### 3.4. Intra-LC Enkephalin Signaling Produces Conditioned Place Preference

**Figure 4:**
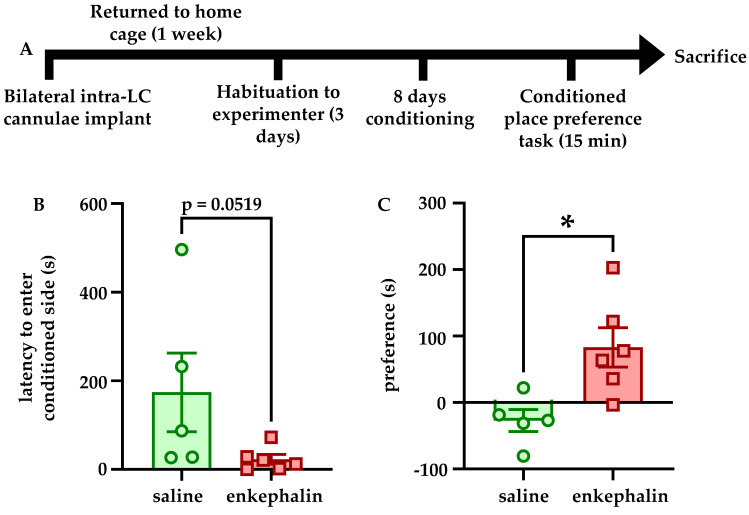
**Intra-LC infusions of enkephalin promote conditioned place preference.** The experimental timeline is shown in (**A**). Rats were habituated to handling by the experimenter for 3 days but were naïve to the behavioral apparatus when conditioning began. Rats that received enkephalin infusions showed a strong trend for entering the conditioned side of the CPP apparatus with shorter latency than the rats that received saline infusions in the conditioned side (*p* = 0.0519, (**B**)). Rats that received enkephalin infusions in the conditioned side of the CPP apparatus showed a significantly greater preference for that chamber than rats that received saline infusions in the conditioned side (**C**). * *p* < 0.05.

To determine if intra-LC enkephalin signaling at DORs affects reward-related learning, rats were subject to a conditioned place preference task according to the experimental timeline shown in Figure 4A. A Mann–Whitney U test showed a trend for significantly reduced latency to enter the conditioned chamber in rats that received enkephalin infusions relative to rats that received saline infusions (U = 4, *p* = 0.0519, Figure 4B). In addition, preference for the conditioned chamber (calculated as the difference between time in the conditioned side and the time in the unconditioned side) was significantly higher for rats that received enkephalin infusions than those that received saline infusions (t = 3.079, df = 9, *p* = 0.132, Figure 4C).

## 4. Discussion

Here, we have shown that acute stressor exposure increases anxiety-like behavior in both the short and long term and decreases DOR expression within LC. Furthermore, LC-specific virally mediated overexpression of DORs blocks the effects of stress on both anxiety-like behavior and LC physiological properties. Finally, intra-LC infusions of enkephalin blocked stress-induced freezing behavior and drove conditioned place preference. These findings indicate that enkephalinergic signaling at DORs within LC both reduces anxiety-like behavior and may be related to reward-related learning. While the role of the pro-stress effector CRF in driving behavior through its actions in LC have been well characterized [8,13,23,30,39], the role of enkephalin, and DORs in particular, has been comparatively understudied. This is an important area for investigation because CRF and endogenous opioids act in concert in LC to respond to a stressor: first, CRF is released onto LC causing depolarization and increased discharge and anxiety-like behavior, and upon stressor termination, endogenous opioids act on their receptors to suppress LC output and facilitate a return to a more normative behavioral state [25,32]. While it has already been established that pharmacological and genetic manipulations of enkephalin and DORs beyond LC affect anxiety-like and motivated behavior [22,34,35,36,37], this is the first study to show that they act within LC specifically to modulate these processes.

One notable finding from this study was that an acute stressor—combined physical restraint and predator odor exposure, which we have previously shown increases anxiety-like behavior and LC firing and excitability for at least a week [18]—also downregulates DOR expression within LC. In addition, the relative quantity of DOR mRNA is significantly positively correlated with center time in the OFT (Figure 1), indicating that reduced DOR levels may contribute to the increased anxiety-like behavior. This may occur because as fewer receptors are available, there is reduced capacity for DOR signaling to decrease LC firing, even in the absence of ligand, as DORs are known to be constitutively active [40]. Such intrinsic or ligand-dependent activation of DORs may reduce LC activity, which has been shown to be causally responsible for anxiety-like behavior [13], and with downregulation of the receptor, LC may become hyperactive. Therefore, it is possible that the increased LC discharge and anxiety-like behavior we have previously identified [18] is due at least in part to stress-induced DOR downregulation in LC. It has previously been shown that CRFR1 activation promotes cellular changes including regulation of transcription [16,17], and thus, one such target in LC neurons may be oprd1, the gene encoding DORs. Notably, we did not find that CRFR1 expression was affected by stress (data not shown). In addition, the fact that viral-genetic overexpression of DORs blocked both the behavioral and physiological effects of stress strongly suggests that this receptor plays an important role in regulating stress susceptibility and resilience.

We also found that Leu-enkephalin infusions directly into LC affected both freezing behavior in response to stress and reward-related behavior in stress-naïve animals. Stressed rats that received enkephalin infusions froze significantly less in the OFT than stressed rats that received saline (Figure 3B). Although there was no effect of enkephalin infusions on center time in the OFT (data not shown), these findings suggest that enkephalin signaling in LC may influence passive coping behaviors such as freezing. It is also possible that the lack of a stress-induced decrease in center time in saline-infused animals is related to the presence of the chronic microinfusion cannulae implants. For example, saline-infused control animals may have spent more time avoiding the center due to the implant relative to non-implanted controls, and stress instead manifested in implanted animals as increased freezing.

Enkephalin infusions into also LC promoted conditioned place preference for the conditioned chamber relative to rats that received saline infusions on both sides of the behavioral apparatus. Although these experiments did not take into account differences in preference for the conditioned chamber in pre- and post-tests, they nonetheless indicate that enkephalinergic signaling within LC affects behavior in the CPP task in some capacity. It is unclear whether the apparent preference for the enkephalin-paired chamber reflects rewarding properties of enkephalin within LC or an attenuation neophobia of the novel conditioned environment during conditioning. For example, when animals were allowed to freely explore the behavioral apparatus, the increased time in the conditioned chamber may actually reflect a reduced aversion for that area relative to the novel unconditioned side. Future studies are necessary to clarify if enkephalin exerted these behavioral effect through reward or through reduction of anxiety-like behavior. It is also important to acknowledge that although we did not explore the effects of stress on MORs, they are in fact targeted by enkephalin and potently inhibit LC neurons as well. Although we did find here that DOR expression is regulated by stressor exposure, and that DOR overexpression itself is sufficient to prevent stress-associated behavioral and physiological phenotypes, further studies are needed to isolate what role MORs in the LC may play in these phenotypes. This is particularly important because MORs and DORs are known to heterodimerize in LC [41], and thus it is not clear if such a downregulation of DORs affects MOR function, and each receptor subtype has specific roles in behavioral responding [31].

Although these studies did not specifically investigate if the enkephalinergic projection to LC becomes engaged in animals that show reduced anxiety-like behavior in response to stress, previous studies have provided indirect evidence that the CRF-positive and enkephalinergic projections to LC become engaged under specific circumstances and aid in driving particular behavioral phenotypes [30]. Specifically, there is evidence for increased activation of the CRF-positive CeA→LC circuit in animals that are stress-susceptible passive copers, and increased activation of the enkephalinergic PGi→LC circuit in animals that are stress-resilient active copers. These findings are important because the persistence of anxiety-like behavior in the absence of an active stressor and the transition from an active stress coping strategy to a passive coping strategy are associated with the transition from a normal adaptive stress response to a maladaptive one in several disease states such as major depressive disorder and generalized anxiety disorder [42,43]. The relative balance of the CRF-containing CeA→LC pathway and enkephalinergic PGi→LC pathways and the potency of each may contribute to a particular behavioral phenotype. For example, animals with a hyperactive CeA→LC and hypoactive PGi→LC projection may display unusually high stress susceptibility and anxiety-like behavior when faced with a stressor. Artificially increasing the activity of the PGi→LC circuit may then drive an animal towards a behavioral phenotype characterized by stress resilience.

It is also notable that LC efferents also seem to encode similar behavioral profiles. Despite early studies showing that LC is a relatively homogeneous entity with highly divergent axons broadly innervating vast expanses of the central nervous system, in recent years, a more nuanced view of the functional organization of LC has come into view. Discrete modules of LC cells have been shown to innervate preferred terminal fields [44,45] and are involved in a number of discrete behaviors including fear conditioning and extinction [46], spinal nociception [47], and exploration [48]. In addition to the anxiogenic role for the CeA→LC circuit, the reciprocal LC→CeA projection serves a similar behavior function. Previous observations from our laboratory have shown that chemogenetic stimulation of the LC→CeA pathway promotes anxiety-like behavior, and inhibition suppresses it [49]. This indicates that there is a positive feedback loop between these two structures to drive anxiety-like behavior during periods of stress. On the other hand, in addition to the stress resilience-promoting PGi→LC circuit, data from our laboratory show that the LC→mPFC projection promotes behaviors opposite to the LC→CeA projection. Specifically, enhancing the output of this pathway causes increased exploration of the elevated plus maze, and suppression of it causes increased freezing behavior. This increased exploratory behavior that occurs in response to stimulation of this circuit is consistent with prior reports as well [48]. While it is clear that the CeA→LC and LC→CeA circuits drive anxiety-like behavior and stress susceptibility, and the PGi→LC and LC→mPFC circuits promote stress resilience and exploration, it is not yet known if these structures are organized into discrete modules such that PGi selectively innervates LC cells that project to mPFC, and CeA selectively innervates LC cells which reciprocally project back to LC. Although some circuit mapping of LC afferents and efferents using mono-transsynaptic rabies-based anatomical tracing has been performed in the past [50], this is an open question regarding exploration. Demonstration that these structures do operate as distinct PGi→LC→mPFC and CeA→LC→CeA circuits would show that each LC module represents an anatomical site whose activity can be targeted for experimental or therapeutic purposes to drive particular behavioral phenotypes.

Even in the absence of an anatomical organization in which LC inputs and outputs are modularly structured, the present findings provide compelling evidence for a role for enkephalinergic signaling at DORs within LC in shaping behavior. The fact that anxiety-like behavior correlates with LC DOR expression, which is itself reduced by stressor exposure, indicates that stress and endogenous opioidergic systems within LC are tightly linked. Because the enkephalin/DOR system acts as a brake for the pro-stress CRF system, the downregulation of the former by the latter may promote a physiological condition in which the LC is unable to adequately respond to novel stressors, thereby creating a phenotype of persistent anxiety-like behavior. However, because we have shown that virally-mediated overexpression of DORs in LC precludes both the physiological and behavioral effects of acute stress, these receptors and their endogenous ligand enkephalin represent an important target for the study and treatment of anxiety-like behavior and anxiety disorders. Additionally, the fact that enkephalinergic signaling in the LC drove conditioned place preference indicates that this brain region is a site beyond the canonical mesolimbic dopamine reward circuitry, where opioid receptors may exert rewarding effects. Because stress downregulates DORs in LC and renders LC hyperactive, which is known to be anxiogenic and aversive [13], opioid drugs of abuse that potently inhibit LC may be more rewarding in animals and humans with a history of chronic or traumatic stress because of their ability to reduce the negative affect associated with LC hyperactivity. Indeed, opioid use disorder and anxiety disorders are frequently comorbid [51]. Gaining a more thorough understanding of how stress and endogenous opioidergic circuits and receptors interact in LC may provide important insights into the development and treatment of anxiety and opioid use disorders.

## Data Availability

Data can be obtained from the corresponding author.
